# Neuroticism as a Common Factor in Depression and Anxiety Associated with Multiple Sclerosis—A Systematic Review and Meta-Analysis

**DOI:** 10.3390/ijerph21101264

**Published:** 2024-09-24

**Authors:** Alina Schenk, Cosmin Octavian Popa, Cristiana Manuela Cojocaru, Ștefan Marian, Smaranda Maier, Rodica Bălașa

**Affiliations:** 1 The Doctoral School, George Emil Palade University of Medicine, Pharmacy, Science and Technology, 540142 Targu Mures, Romania; alina.muresan@umfst.ro (A.S.); cristiana-manuela.cojocaru@umfst.ro (C.M.C.); 2 Department of Ethics and Social Science, George Emil Palade University of Medicine, Pharmacy, Science and Technology, 540142 Targu Mures, Romania; 3 Department of Psychology, West University of Timişoara, 4 Vasile Pâvan Boulevard, 300223 Timişoara, Romania; stefan.marian@e-uvt.ro; 4 Neurology Clinic I, Emergency Clinical County Hospital, 540163 Targu Mures, Romania; smaranda.maier@umfst.ro (S.M.); rodica.balasa@umfst.ro (R.B.); 5 Department of Neurology, George Emil Palade University of Medicine, Pharmacy, Science and Technology, 540142 Targu Mures, Romania

**Keywords:** personality, neuroticism, depression, anxiety, multiple sclerosis, fatigue, quality of life

## Abstract

Background: Left undiagnosed and untreated, the association between multiple sclerosis and mental health difficulties significantly increases the multimorbidity risk in these patients. Hence, the purpose of this systematic review and meta-analysis was to estimate the prevalence of neuroticism, depression, and anxiety in MS and to explore the cumulative impact of these psychological factors on the disease expression. Methods: A literature search was conducted on PubMed, Web of Science, Scopus, and Google Scholar databases, according to the PRISMA guidelines. Also, the potential risk of bias was assessed using the AXIS tool. Result: After a rigorous full-text examination, among the 756 identified studies, 22 investigations were considered for the systematic review, and 10 studies were selected for the meta-analysis. The prevalence of neuroticism in the studied population was 24.06% (95% CI: 16.79–33.34), of depression 20.77% (95% CI: 7.67–33.88), while the presence of anxiety was found in 23.94% (95% CI: 6.21–40.36). Conclusions: The main finding of this research confirms that psychiatric disorders often co-occur with MS, impacting the clinical symptoms and life quality of patients living with this illness. For a better understanding of the interaction between personality, depression, anxiety, and the disease symptoms, future research should consider conducting comparisons on more homogenous studies.

## 1. Introduction

Among neurological diseases, multiple sclerosis (MS) is by far the most debilitating in youth, its onset taking place between the ages of 20 and 40 [1]. Thus, over the last 20 years, there has been an extraordinary progression related to the diagnosis and treatment of this neurodegenerative illness, its clinical manifestations negatively impacting the patient’s autonomy, employment security, interpersonal relations, and overall quality of life. Therefore, the most frequent complaints of patients with MS include visual problems, walking difficulties, fatigue, neuropathy, sensorial discomfort, intestinal and bladder dysfunctions, infections, cognitive impairment, as well as psychological disturbance [2,3]. In addition, a preoccupation of recent studies was directed toward the disease onset at an older age, especially due to a more progressive form of MS, motor dysfunctions being the most common symptom in these patients [4,5]. The challenges encountered by the specialists in the diagnosis and treatment of this category of patients are determined by the increased prevalence of comorbidities and age-specific symptoms [6]. At the same time, throughout the course of the disease, depression and anxiety disorders are the most prevalent psychiatric conditions. Also, changes in personality, such as apathy, social isolation, impulsivity, and decreased empathy levels, were observed in patients with MS [7,8]. These modifications in the personality of patients with MS could be owed to the presence of diverse agents such as brain lesions, medication, psycho-social factors, and pre-existing mental health conditions [7].

Personality refers to the individual’s pervasive and permanent pattern of thoughts, feelings, and behaviors expressed in diverse personal and social contexts. Furthermore, personality traits are related to functional or dysfunctional responses in stressful situations, which explains interindividual differences. Therefore, it is highly important to identify personality characteristics of patients with MS to anticipate their response to the disease progression. Moreover, dysfunctional personality traits and personality disorders were related to the occurrence of psychopathology and were found to predict the relapse of major depressive disorder [9,10,11]. The Five Factor Model (FFM) of personality is considered one of the most reliable personality assessment tools comprising five central traits: openness, extraversion, consciousnesses, agreeableness, and neuroticism [12]. Openness is related to artistic, intellectual, and curiosity tendencies. Extraversion is associated with high levels of energy and the need for social stimulation. Consciousnesses correlate with discipline, performance, and a high sense of duty, while agreeableness describes peaceful and empathetic individuals oriented to help others in need. Neuroticism is characterized by the proneness to experience intense negative emotional reactions in stressful situations like anxiety, anger, and sadness accompanied by the difficulty in regulating this overwhelming emotional response [13]. According to Widiger, neuroticism represents the main factor involved in mental health disorders, hampering the public health systems because of its association with multiple medical conditions [14]. The prevalence of dysfunctional patterns of personality associated with MS was the interest of multiple investigations. The outcomes of these investigations showed that neuroticism is the most widespread dysfunctional personality trait in patients with MS, predicting a lower adherence to the standard treatment of this illness, the use of maladaptive coping strategies for facing clinical symptoms, and worsening the disease progression [15,16,17]. Moreover, neuroticism represents a strong predictor of recurrent affective and anxiety disorders [18,19]. Given that this personality trait increases the likelihood of engaging in unhealthy cognitive and behavioral strategies, like worries, ruminations, and avoidance, it contributes to the onset of these psychological difficulties.

Based on this empirical background, the aim of this review is to assess the co-occurrence between neuroticism as the most representative trait of dysfunctional personality, depression, and anxiety in patients with MS, along with their cumulative impact on other clinical outcomes. We hypothesize that patients with MS presenting a higher neuroticism level would also have an affective psychodiagnosis. In addition, knowing that personality dysfunction and psychopathology are highly prevalent in this disease, our second hypothesis states that patients with MS presenting a dysfunctional personality intertwined with anxiety and depression would also report increased levels of fatigue, cognitive impairment, as well as diminished health-related quality of life.

## 2. Materials and Methods

The present systematic review was elaborated according to the revised Preferred Reporting Items for Systematic Reviews and Meta-Analysis (PRISMA) 2009 checklist. Also, a meta-analysis was conducted only on those studies that reported the results on neuroticism and emotional disorders using the same descriptive statistics, specifically means and standard deviations. The meta-analysis for one group mean was performed using the rBiostatistics.com (accessed on 28 June 2024) cloud-based statistical software [20]. All studies included in this present review were assessed from the perspective of risk of bias using the assessment tool for observational studies named AXIS [21].

Studies included in this systematic review and meta-analysis were identified by searching PubMed, Web of Science, Scopus, and Google Scholar. The following terms were used for identifying studies between 2012–2023: “personality “AND “personality trait” AND “personality disorder”, “depression” AND “depression disorder”, “anxiety “AND “anxiety disorder”, “emotional disorder”, “multiple sclerosis “AND “MS”. The inclusion criteria were: (1) final diagnosis of MS according to McDonald criteria, as established by a neurologist, (2) assessment of personality, (3) assessment of depression and/or anxiety, (4) patients aged ≥ 18 years old, (5) studies published in peer-review journals, (6) English language, (7) cross-sectional studies, case–control studies, observational studies. The exclusion criteria were: (1) patients diagnosed with associated severe neurological conditions, (2) patients with severe psychiatric disorders such as psychotic disorders, schizophrenia, and bipolar disorder (3) case studies, (4) minor patients.

The study selection was performed in a step-wise manner. Therefore, in the first stage, after eliminating the duplicates, two authors analyzed the studies in terms of title and abstract. A third author was invited to analyze those articles on which the other two authors were unclear about eligibility. In the following stage, the researchers read all remaining articles in full-text and completed data collection to reduce the risk of bias (i.e., diagnosis, variable assessment, assessment data reporting, patients’ selection). The overall level of agreement between the first two researchers was good, as reflected by the obtained Cohen’s k = 0.70.

## 3. Results

### 3.1. Studies Characteristics

**Figure 1:**
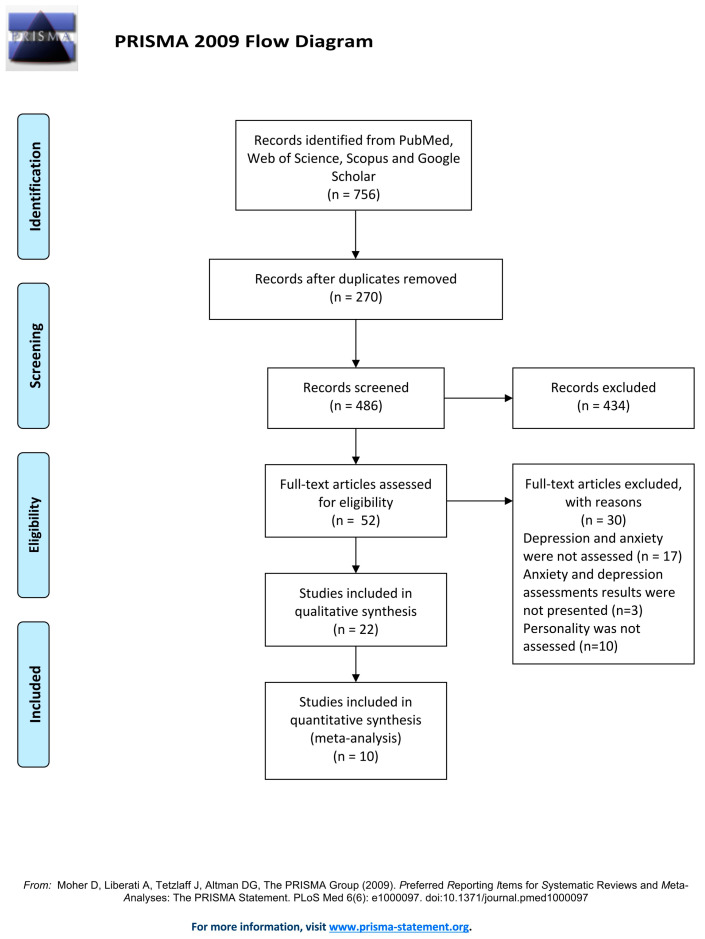
The search strategy flow diagram.

Data were extracted from 756 studies. After excluding duplicates, 486 studies were screened and the full texts of the remaining 52 were analyzed for eligibility (Figure 1). The database included the following information: authors, year of publication, participant characteristics (age and sex), selection method, and assessments (anxiety, depression, personality, and other variables). After a thorough evaluation conducted by the involved authors, twenty-two studies that met the inclusion criteria were included in the systematic review. The studies explored the impact of personality traits on different outcomes, such as depression, anxiety, fatigue, cognitive and physical function, employment status, disability, and quality of life. In terms of personality measurement, fifteen studies used self-reported inventories based on the FFM of personality. Specifically, three studies used two different self-reported editions of the Millon Clinical Multiaxial Inventory (MCMI), two studies applied the self-reported Type D Scale-14 (DS-14), and one study used the self-reported Eysenck Personality Questionnaire Revised-Short Form (EPQ-RS). Only one study assessed personality by conducting a semi-structured interview applying the Structured Clinical Interview for DSM, Revised Third Edition Personality Disorders (SCID-III-PD). As for the psychopathology assessment, all twenty-two investigations measured depression, while fifteen also included measures of anxiety. All studies reported the use of adapted and validated instruments to measure the severity of depression and anxiety. The selected studies were conducted in the following countries: Slovakia, Iran, USA, Germany, The Netherlands, Italy, Spain, Republic of Korea, Turkey, UK on samples ranging from 24 to 384 MS patients. Table 1 presents the most important characteristics of the included studies. The relationship between personality and psychopathology was the main objective of fifteen studies [22,23,24,25,26,27,28,29,30,31,32,33,34,35,36], while in seven studies, personality was explored directly in relation to other variables like fatigue, cognition, employment, and quality of life [37,38,39,40,41,42,43].

### 3.2. Risk of Bias Assessment

The risk of bias for all the studies included in this paper was analyzed using a tool designed for observational studies that includes the critical appraisal of the introduction, methodology, results, and discussion sections. Thus, 20 questions were addressed for each study in order to assess their quality by answering with “yes”,” no” or “don’t know”. Based on the AXIS assessment, we found that all investigations used appropriate study designs in concordance with the reported objectives. Regarding the sample size requirements, we identified only one study that reported sample size calculation [27], while the selection process for the included participants was clearly described for ten studies [22,24,25,27,29,38,39,40,41,43]. Eight studies included covariates in their statistical analysis, providing more robust findings [23,24,25,28,33,37,40,43]. We did not assess the non-responder rate because all studies reported complete data. The majority of investigations included comprehensive details of the results, limitations, and conclusions. All studies conducted the research after obtaining the informed consent of the participants.

### 3.3. Neuroticim Co-Occurring with Depression

As the most prevalent psychiatric disorder in MS, depression was assessed in all selected studies, along with personality traits. While some instruments used in the measurement of depression focused on the diagnosis of depressive disorder, others emphasized the evaluation of depressive symptomatology. Several studies explored the associations between personality, depression, and cognitive functions [21,28,38,39,40,42]. Elevated neuroticism was directly proportional to depression, whereas higher extraversion positively correlated with better self-reported cognitive functioning [28]. Maggio et al. explored cognitive rehabilitation in patients with MS, and found that neuroticism was directly linked to poor attention and increased memory dysfunction. At the same time, neuroticism represented the main personality trait involved in the onset of depressive symptomatology within the MS sample [38]. Raimo et al. found no association between depression, neuroticism, and prospective memory deficits in a sample comprising 33 patients with MS. In contrast, lower extraversion and openness were predictors of prospective memory impairment [39]. As compared with healthy controls, patients with MS reported higher levels of depression, cognitive errors, and neuroticism [40]. The association between depressive mood and neuroticism in patients with MS was also investigated in relation to fatigue, one of the most burdensome symptoms in patients with MS. Thus, compared with healthy controls, in patients with MS, increased depression, fatigue, and neuroticism levels correlated with lower gray matter volume [25]. Together with depression, neuroticism was a strong predictor of cognitive fatigue in patients with MS [27,31,38], while lower levels of extraversion were related to physical fatigue [28,34]. Furthermore, several studies included quality of life as an outcome of the interplay between personality and depression in their investigation. As hypothesized, patients who scored higher on negative personality traits like neuroticism and depression scored lower on health-related quality of life [23,24,32,34,36,38,41]. Three analyses explored the presence of personality disorders and other psychopathologies in patients with MS. Their outcomes emphasized the predictive role of personality dysfunctional patterns in the occurrence of affective disorders in patients with MS compared with controls [27,31,35]. Although increased scores of neuroticism were observed, the occupational status of MS patients was negatively linked with depression and fatigue but not with personality dysfunctional traits [42].

### 3.4. Neuroticism Co-Occurring with Anxiety

Fifteen studies included anxiety as the dependent variable in relation to personality. A higher prevalence of anxiety was observed in newly diagnosed MS patients who reported accentuated neuroticism, along with diminished extraversion, consciousness, and agreeableness [22]. Moreover, in conjunction with increased neuroticism and lower extraversion, anxiety significantly predicts the deterioration of both physical and mental quality of life [23,24,38,41]. In their research, Ghahremani et al. confirmed the elevated prevalence of anxiety in patients with MS compared to controls, which had a further negative impact on their quality of life. Generalized anxiety disorder, panic attacks, and somatization syndrome are often associated with personality disorders in MS, influencing disease progression and relapses [31,32,35]. Additionally, patients with higher levels of neuroticism and lower levels of extraversion showed exacerbated anxiety. In combination with increased fatigue levels, anxiety is also correlated with work and information processing difficulties in patients with MS [42,43].

### 3.5. Meta-Analysis

**Figure 2:**
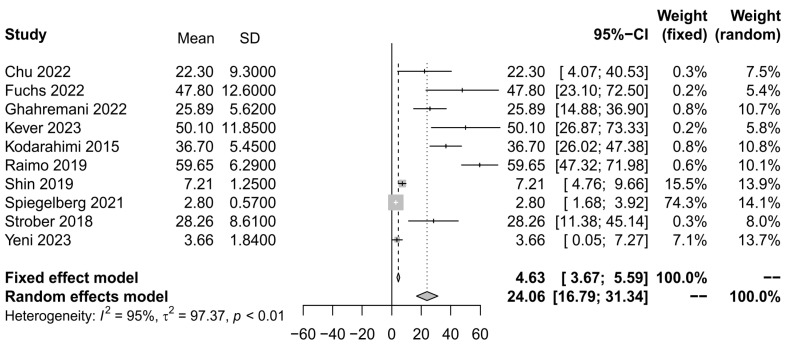
Forest plot of studies for neuroticism, with a 95% confidence interval.

Ten studies [22,25,26,29,32,36,39,40,41] reported data in a way that allowed us to conduct a meta-analysis to evaluate the effect of neuroticism as a main factor contributing to psychopathology in patients with MS. Using the random effects model, the meta-analysis summary showed a prevalence of neuroticism of 24.06 (95% CI: 16.79–33.34), z = 6.48, *p* < 0.0001. As expected, heterogeneity measured by *I*^2^ was 92%, t^2^ = 97.37, *p* < 0.01, indicating an increased variability in the outcomes. The size of the square indicates the weight of the study. Thus, only one study seems to have a larger effect size for the outcomes obtained (Figure 2).

**Figure 3:**
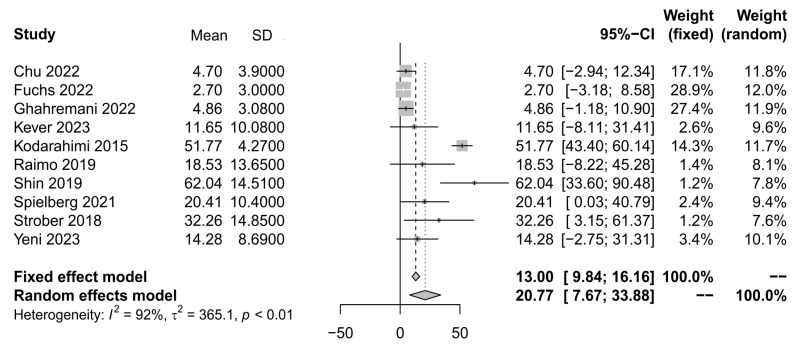
Forest plot of studies for depression, with a 95% confidence interval.

To calculate the prevalence of depression in patients with MS, we included the same ten studies [22,25,26,29,32,36,39,40,41]. Given the increased heterogeneity, we report a random effects model of 20.77 (95% CI: 7.67–33.88), z = 3.11, *p* < 0.019, *I*^2^ = 92%, t^2^ = 365.1, *p* < 0.01). A larger effect size for depression outcomes was found in three studies (Figure 3).

**Figure 4:**
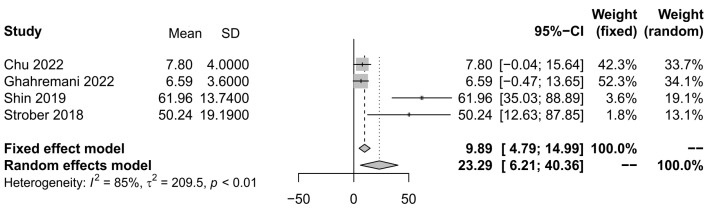
Forest plot of studies for anxiety, with a 95% confidence interval.

Only four studies [22,26,32,41] used measures of the prevalence of anxiety, which allowed the examination of the results. Considering the heterogeneity of the studies, a random effects model of 23.94 (95% CI: 6.21–40.36), z = 2.67, *p* < 0.0075, *I*^2^ = 85%, t^2^ = 209.5, *p* < 0.01 was used. The outcomes of the two studies appear to have larger effect sizes on anxiety (Figure 4).

The increased heterogeneity of the studies included in the present meta-analysis could be attributed to the diversity of the used instruments. Thus, depression was measured by applying seven different types of self-reports. To evaluate anxiety, three types of scales were used, and neuroticism was measured using five distinct personality inventories.

## 4. Discussion

The major finding of this present review was the association between neuroticism as a dysfunctional personality trait, psychopathology, and the clinical symptoms of MS. Moreover, the outcomes of the meta-analysis revealed a comparable prevalence of neuroticism, depression, and anxiety in the MS population. Our results are similar to those reported by other systematic reviews and meta-analyses on the prevalence of anxiety and depression in patients with MS. After analyzing 118 studies, Marrie et al. confirmed the existence of an elevated prevalence of psychiatric comorbidities among patients with MS [44]. Moreover, after exploring the presence of psychopathology-indifferent subgroups of patients with MS, Peres et al. concluded that the prevalence of depression and anxiety was higher in patients with lower disability and Progressive MS type [45]. In their scoping review, Maggio et al. underlined the increased prevalence of dysfunctional personality in patients MS compared to that in the general population. Moreover, they emphasized the negative impact of dysfunctional personality traits on cognition, mood, fatigue, and the overall quality of life [46]. In the same way as in previous research, the heterogeneity is high among the studies included in our meta-analysis, imposing a cautious interpretation of our outcomes. Some causal factors could be considered, such as the diversity of measurements, other methodological differences, patient characteristics, or different study designs.

As a complex and uncurable neurodegenerative disease affecting the central nervous system, MS is often associated with other conditions like autoimmune [47], cardiovascular diseases [48], and psychiatric disorders [44]. This co-occurrence was shown to have considerable implications for the clinical course of the illness and its socioeconomic consequences [49]. Furthermore, the high prevalence of comorbidities is strongly correlated with an increased rate of multimorbidity in patients with MS [50]. As highlighted in this review, the last decade of research on the links between personality and emotional disorders reinforces the role of neuroticism as a salient etiological factor involved in the development of common mental health disorders in comorbidity with MS. Therefore, proper assessment of personality and psychopathology, using both self-reported instruments and structured clinical interviews, is highly recommended starting from the onset of the illness. Moreover, clinicians should also consider evaluating other factors involved in the onset of psychiatric disorders, like dysfunctional psychological mechanisms and psychological processes, which were identified as mediators of the interplay between dysfunctional personality traits and clinical symptoms in MS and other chronic medical conditions [51,52]. This important phase would involve the development of personalized interventions applied within a multidisciplinary approach to enhance disease management in MS. Furthermore, considering the significant role played by the Patient Support Program (PSP) on MS patients’ adherence to treatment [53], our findings could alleviate the burden of this illness through the development of prevention programs aimed at building knowledge and coping skills for the early management of emotional distress associated with MS. Ultimately, our results are consistent with the model of negative affectivity, emphasizing that neuroticism is a hallmark of both personality and emotional disorders in the general population [54]. Based on these findings, we assume that these relationships are also encountered in MS.

The present systematic review and meta-analysis had some methodological limitations. First, we included studies that assessed personality, depression, and anxiety at different times after the onset of the disease. Therefore, outcomes could be hindered by other factors related to the clinical symptoms of MS. Future meta-analyses should consider studies exploring potential confounding factors like medication, comorbidities, or sociodemographic status, all of which could influence the psychological well-being of patients. Second, because of the small number of included studies, the conduction of a thorough subgroup analysis based on the use of specific instruments for the assessment of the pursued variables was difficult. Third, an increased level of heterogeneity represents another constraint in our analysis. For this reason, the results should be carefully considered. In this way, future systematic reviews should include studies that apply longitudinal assessments to emphasize the association between neuroticism and psychopathology.

## 5. Conclusions

This systematic review and meta-analysis emphasized the interconnectedness determined by the clinical expression of psychiatric comorbidities in the evolution and management of MS. By analyzing the included investigations, we outlined an increased prevalence of neuroticism, depression, and anxiety in this population. However, due to the heterogeneous nature of the studies, the reliability of these results could be impeded. Therefore, further analyses should consider extensive comparisons between studies with more homogenous study designs and methodologies. In addition, including covariate exploration could optimize the coherence of outcomes. Nonetheless, an accurate assessment of personality and emotional disorders would facilitate a personalized treatment approach for patients with MS.

## Figures and Tables

**Table 1:** Characteristics of included studies.

Authors, Publication Year	Study Design	N	Selection Method	Age (M/SD)	Female %	Measurements			
						Anxiety	Depression	Personality	Others
Chu et al., 2022 [22]	Retrospective	384	MS clinic	37.8 ± 10.4	68.8	HADS	HADS	NEO-FFI	Cognitive function
Demirci et al., 2017 [23]	Cross-sectional	74	Outpatient clinic	35.3 ± 7.00	63.51	BAI	BDI	DS-14	Quality of life
Dubayova et al., 2012 [24]	Cross-sectional	198	Clinical database	38.4 ± 10.8	67.7	HADS	HADS	DS-14	Disease severity, health-related quality of life
Fuchs et al. 2022 [25]	Case-control	137	NP	53.2 ± 11.6	72.3	-	BDI-FS	NEO-FFI	Fatigue, cognition, disability, self-report severity of cognitive dysfunction
Gharemanti et al. 2022 [26]	Cross-sectional Case–control	100	Patients from clinical settings	37.29± 9.7	73.27	HADS	HADS	NEO-FFI	-
Incerti et al., 2015 [37]	NP	77	Neurology unit	43.1 ± 9.8	56	STAI	CMDI	MCMI III	Fatigue
Kamal et al., 2018 [27]	Correlational	88	Psychotherapeutic center	33.03 ± 9.032	63.3	MCMI-II	MCMI-II	MCMI-II	-
Kever et al., 2023 [28]	Cross-sectional	129	University medical center	45.7 ± 10.9	76.0	-	BDI II	NEO-FFI	Cognitive and physical functions
Khodarahimi et al., 2015 [29]	Quasi-experimental	30	Outpatient clinics	27.95 ± 2.43	0	-	BDI	NEO-PI-R	Fatigue
Lima et al., 2014 [30]	Cross-sectional	33	outpatient Neurology service	36.96 ± 9.57	100	BAI	BDI	NEO-FFI	-
Maggio et al., 2022 [38]	Exploratory study	31	Neurorehabilitation clinic	50 ± 10.7	55	HRS-A	BDI II	Big five Questionaire	Cognitive function, quality of life
Mirzaei et al., 2012 [31]	Case-control	94	Neurological clinics	33.03 ± 8.9	76.6	MCMI-II	MCMI-II	MCMI-II	Clinical syndrome
Raimo et al., 2019 [39]	NP	33	MS center	44.60 ± 13.02	60.6	-	BDI II	NEO-P-I-3	Cognitive function, apathy
Shin et al., 2019 [32]	Observational	24	Neurology department	32.13 ± 11.82	70.8	SCL-95	SCL-95	BFI-K-10	Hopelessness, quality of life, Positivity offset, negative bias, heart rate variability
Sinderman et al., 2018 [33]	Cross-sectional	52	Neurological rehabilitation center	45.13 ± 9.56	82.69	-	ADS	NEO-FFI	Fatigue
Spielberg et al., 2021 [40]	Case-control	30	Neurological department	46.1 ± 9.6	61.76	-	CES-D	NEO-FFI, ANPS	Cognitive deficits, fatigue
Strober et al., 2018 [41]	Longitudinal	64	NP	NP	89.85	STAI	CMDI	NEO-FFI	Fatigue, quality of life, coping to stress
Strober, 2017 [34]	Longitudinal	233	NP	NP	85.4	STAI	CMDI	NEO-FFI-3	Fatigue, pain, sleep, disease management and self-efficacy, locus of control
Uca et al., 2016 [35]	Cross-sectional	55	Neurology clinic	34.07 ± 8.16	85.5	SCID-I/CV	SCID-I/CV	SCID-II-PD	Disability
van der Hiele et al., 2020 [42]	Case-control	278	Outpatients clinics	43 ± 14.0	78	HADS	HADS	NEO-FFI	Empathy, disability, cognitive function, occupational status
van der Hiele et al., 2021 [43]	Case-control	241	Outpatient clinics	42 ± 14.0	78.0	HADS	HADS	NEO-FFI	Fatigue, occupational status, disability, fatigue, cognitive function
Yeni et al., 2023 [36]	Case-control	80	Outpatient clinic	43.01 ± 11.22	66.3	-	BDI II	EPQ-RS	Stigmatization, quality of life

Note: N = sample size, M = mean, SD = standard deviation, NP = not precised, HADS = Hospital Anxiety and Depression Scale, DS-14 = Type D Scale-14, BDI-FS = Beck Depression Inventory-Fast Screen, NEO-FFI = NEO-Five Factor Inventory, MCMI-II = Millon Clinical Multiaxial Inventory II, BDI = Beck Depression Inventory, NEO-PI-R = NEO-Personality Inventory-Revised, CES-D = epidemiologic studies-depression scale, ANPS = affective neuroscience personality scale, BDI II = Beck Depression Inventory -II, EPQ-RS = Eysenck Personality Questionnaire Revised-Short Form, SCID-I/CV = Structured Clinical Interview for the Diagnostic and Statistical Manual of Mental Disorders, Fourth Edition/Clinical Version, SCID-II = Structured Clinical Interview for DSM, Revised Third Edition Personality Disorders, STAI = The State Trait Anxiety Inventory, CMDI = Chicago Multiscale Depression Inventory, NEO-P-I-3 = NEO-Personality Inventory-3, SCL-95 = Symptom Checklist-95, BFI-K-10 = Big Five Inventory-Korean-10, HRS-A = Hamilton Raiting Scale Anxiaty, MCMI III = Millon Clinical Multiaxial Inventory III, NEO-FFI-3 = NEO-Five Factor Inventory-3, BAI = Beck Anxiety Inventory, ADS = Allgemeine Depressionsskala.

## Data Availability

The data presented in this study are available upon request from the corresponding author (cosmin.popa@umfst.ro).
